# LB1578. Fully Automated Real-Time PCR for Detection of *Candida auris*

**DOI:** 10.1093/ofid/ofac492.1888

**Published:** 2022-12-15

**Authors:** Alice Jiang, Mike Lin, Nesreen Barakat, Craig Clark

**Affiliations:** Hologic, San Diego, CA; Hologic, San Diego, CA; Hologic, San Diego, CA; Hologic, San Diego, CA

## Abstract

**Background:**

*Candida auris* is an emerging fungus that presents a serious global health threat. It is often multidrug-resistant and difficult to identify with standard laboratory methods. *C. auris* outbreaks have been associated with high mortality in healthcare settings. Rapid and accurate screening for *C. auris* is crucial for preventing transmission and directing treatment. This study evaluated a new fully automated, lab-developed real-time PCR assay for the detection of *C. auris*.

**Methods:**

Nasal swabs collected with Aptima Multitest swabs were used as a representative clinical matrix to evaluate *C. auris* detection using the Open Access™ functionality on the Panther Fusion® instrument. It provided fully automated nucleic acid extraction, amplification, and detection workflow. The primers and probe are published sequences designed by CDC which target the internal transcribed spacer 2 (ITS2) region of the *C. auris* ribosomal RNA gene. Analytical studies evaluated assay sensitivity, specificity, and inclusivity. An internal control is also included to ensure assay validity.

**Results:**

The limit of detection (≥ 95% detection rate) of the *Candida auris* real-time PCR assay is 5 CFU/mL for nasal swabs. Eleven *C. auris* strains from CDC clinical isolates were reactive for inclusivity study. No cross reactivity or interference was observed from 60 microorganisms of closely related yeast, bacteria and viruses typically present in skin and groin samples.

**Conclusion:**

A fully automated and high throughput real-time PCR test for *C. auris* with internal control, utilizing primers and probe sequences published by CDC, demonstrated excellent sensitivity, specificity, and inclusivity. This lab developed test (LDT) provides a rapid, sensitive, and scalable method for detection of *C. auris.* The results are crucial for timely detection, transmission prevention and targeted treatment of the multidrug-resistant *C. auris.*

**Disclosures:**

**All Authors**: No reported disclosures.

